# Composition by Sex of State and Federal Legislative Health Committees

**DOI:** 10.1001/jamanetworkopen.2026.13624

**Published:** 2026-05-20

**Authors:** Daeun Sung, Michael K. Rooney, Latifat Sulaimon, Reshma Jagsi, Fumiko Chino, Nancy Spector, Gita Suneja, Brielle Shorter, Angela Fagerlin, Kekoa Taparra, Mackenzie Nielson, Bismarck C. Odei

**Affiliations:** 1 Frank H. Netter MD School of Medicine, Quinnipiac University, North Haven, Connecticut; 2 Department of Radiation Oncology, MD Anderson, Houston, Texas; 3 Ohio State University, Columbus; 4 Department of Radiation Oncology, Emory University, Atlanta, Georgia; 5 Department of Pediatrics, Drexel University College of Medicine, Philadelphia, Pennsylvania; 6 Department of Radiation Oncology, Huntsman Cancer Institute, University of Utah, Salt Lake City; 7 Department of Population Health Sciences, Spencer Fox Eccles School of Medicine, University of Utah, Salt Lake City; 8 Department of Radiation Oncology, University of California, Los Angeles

## Abstract

This cross-sectional study examines the composition of state and federal legislative health committees by sex.

## Introduction

State and federal legislation shape US health policy, and much of this work occurs within legislative health care committees, where bills are introduced, amended, rejected, or advanced to the full chamber for debate and voting.^1^ Given these committees’ impact on policy priorities, understanding their demographic composition offers insight into potential influences on health policy decision-making.^2,3,4^

## Methods

This cross-sectional study followed the STROBE reporting guideline and was deemed exempt by the University of Utah institutional review board. In August 2023, data on state and federal legislators and committees were obtained from KnowWho,^5^ a commercial database of government officials used in prior research.^6^ Health care committees were identified by manual review of all state and federal legislative committees and classified as committees whose function involved the maintenance and improvement of physical and mental health through the prevention, diagnosis, treatment, and management of disease, as well as policies and programs for the promotion of health. Classification was independently determined by 2 authors, with a third author serving as tie-breaker. Race and ethnicity were obtained from the database, rather than assigned by investigators, and were categorized as American Indian or Native American, Asian or Pacific American, Black or African American, Hispanic or Latino, Native Hawaiian or Pacific Islander, White or Caucasian, 2 or more ethnicities, and other.

Categorical variables were compared using χ^2^ or Fisher exact tests and continuous variables using Kruskal-Wallis tests; all tests were 2-sided, with *P* < .05 indicating statistical significance. Data were analyzed from February to March 2025 using R version 4.4.3 (R Project for Statistical Computing).

## Results

A total of 4524 committees were identified, with 8058 state and federal legislative members, including 2458 female and 5060 male legislators at the state level and 154 female and 386 male legislators at the federal level. Of these, 404 committees (8.9%) were classified as health care committees, including 372 state committees and 32 federal committees.

Female legislators were less likely to serve on state-level health care committees than on state-level nonhealth care committees (1109 of 2577 [30.1%] vs 1349 of 2483 [35.2%]; *P* < .001), but were not significantly different in their participation on both committee types at the federal level. Most health care committees were majority male: 255 state committees (68.5%) and 28 federal committees (87.5%) had less than 50% female membership, and some federal committees had no female members (Figure).

**Figure.**
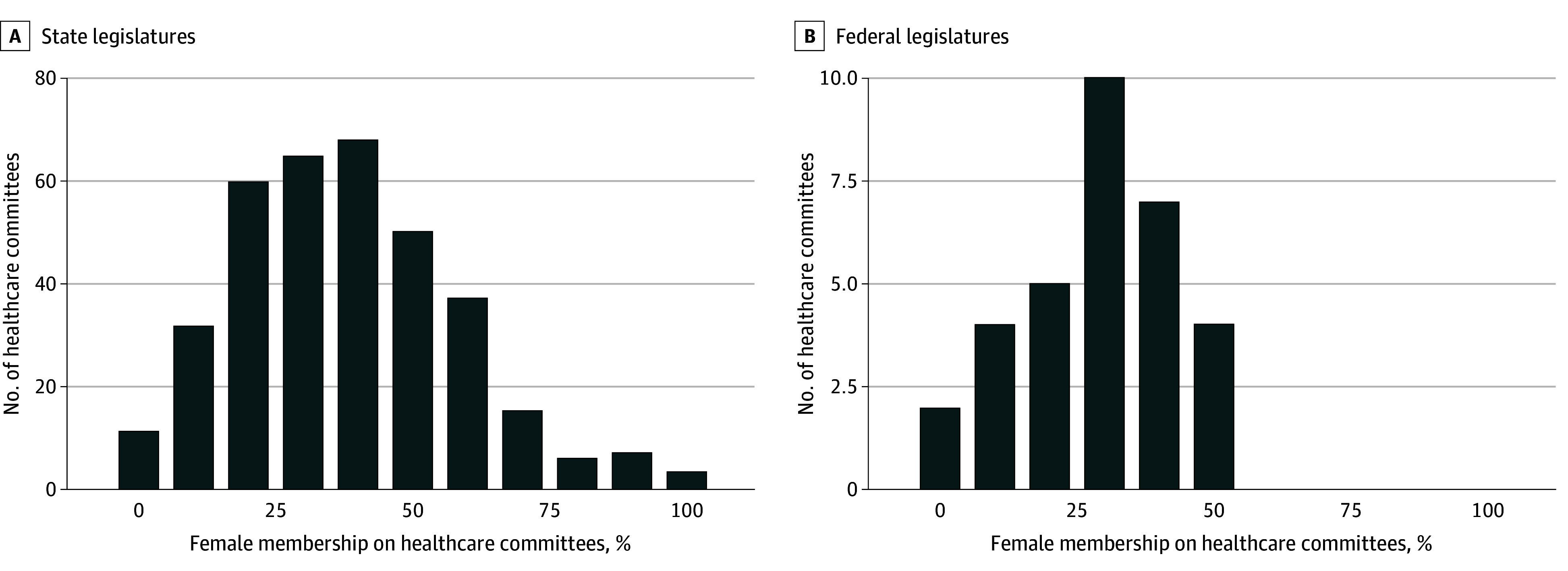
Bar Graph Showing Number of Health Care Committees Among State (A) and Federal (B) Legislatures Stratified by Percentage of Female Legislators

Among health care committee members (Table), female legislators were more likely than male legislators to serve in the House of Representatives at both the state level (985 of 1349 [73.0%] vs 1697 of 2483 [68.3%]; *P* = .003) and federal level (56 of 71 [78.9%] vs 125 of 199 [62.8%]; *P* = .02) and were more recently elected at both levels (state, 456 of 1349 [31.5%] vs 655 of 2483 [26.4%]; *P* < .001; federal, 15 of 71 [21.1%] vs 34 of 199 [17.1%]; *P* = .009). Female legislators were also more likely to have a Democratic party affiliation (state, 852 of 1349 [63.2%] vs 795 of 2483 [32.0%]; *P* < .001; federal, 43 of 71 [60.6%] vs 65 of 199 [32.7%]; *P* < .001), less likely to be married (state, 875 of 1349 [64.9%] vs 1930 of 2483 [77.7%]; *P* < .001; federal, 51 of 71 [71.8%] vs 178 of 199 [89.4%]; *P* = .003), and more likely to belong to racial or ethnic groups underrepresented in government (state, *P* < .001; federal, *P* = .006). At the state level, female legislators were more likely than male legislators to hold graduate degrees (553 of 1349 [41.0%] vs 827 of 2483 [33.3%]; *P* < .001) and to have professional backgrounds in public service (919 of 1349 [37.4%] vs 1095 of 2483 [30.5%]; *P* < .001) or health care (158 [6.4%] vs 139 [3.9%]; *P* < .001). At the federal level, educational attainment and professional background did not differ by sex.

**Table.** Characteristics of State and Federal Legislative Health Care Committee Members

Characteristic	State			Federal		
	Legislator, No. (%)		*P* value	Legislator, No. (%)		*P* value
	Female (n = 1349)	Male (n = 2483)		Female (n = 71)	Male (n = 199)	
Chamber						
House of Representatives	985 (73.0)	1697 (68.3)	.003	56 (78.9)	125 (62.8)	.02
Senate	364 (27.0)	786 (31.7)		15 (21.1)	74 (37.2)	
% Vote obtained						
Median (range)	63.9 (5.30-100)	71.0 (7.40-100)	<.001	59.5 (0.200-100)	62.9 (48.8-100)	.10
Missing or NA	10 (0.7)	19 (0.8)		NA	NA	
Date elected						
Before 2000	35 (2.6)	79 (3.2)	<.001	7 (9.9)	23 (11.6)	.009
2001-2005	28 (2.1)	68 (2.7)		5 (7.0)	11 (5.5)	
2006-2010	68 (5.0)	171 (6.9)		2 (2.8)	33 (16.6)	
2011-2015	154 (11.4)	400 (16.1)		11 (15.5)	42 (21.1)	
2016-2020	639 (47.4)	1110 (44.7)		31 (43.7)	56 (28.1)	
2021 or later	425 (31.5)	655 (26.4)		15 (21.1)	34 (17.1)	
Party affiliation						
Democrat	852 (63.2)	795 (32.0)	<.001	43 (60.6)	65 (32.7)	<.001
Republican	456 (33.8)	1651 (66.5)		24 (33.8)	133 (66.8)	
Other	41 (3.0)	37 (1.5)		4 (5.6)	1 (0.5)	
Marital status						
Single	39 (2.9)	42 (1.7)	<.001	5 (7.0)	9 (4.5)	.003
Divorced	12 (0.9)	12 (0.5)		6 (8.5)	3 (1.5)	
Married	875 (64.9)	1930 (77.7)		51 (71.8)	178 (89.4)	
Not stated	306 (22.7)	355 (14.3)		2 (2.8)	2 (1.0)	
Other	117 (8.7)	144 (5.8)		7 (9.9)	7 (3.5)	
Racial or ethnic background						
American Indian or Native American	13 (1.0)	11 (0.4)		0 (0)	0 (0)	
Asian or Pacific American	31 (2.3)	29 (1.2)	<.001	2 (2.8)	1 (0.5)	.006
Black or African American	229 (17.0)	259 (10.4)		13 (18.3)	16 (8.0)	
Hawaiian or Pacific Islander	6 (0.4)	9 (0.4)		1 (1.4)	0 (0)	
Hispanic or Latino	90 (6.7)	84 (3.4)		4 (5.6)	17 (8.5)	
White or Caucasian	917 (68.0)	2022 (81.4)		47 (66.2)	162 (81.4)	
Two or more ethnicities	14 (1.0)	12 (0.5)		2 (2.8)	1 (0.5)	
Other^a^	19 (1.4)	21 (0.8)		2 (2.8)	2 (1.0)	
Missing or NA	30 (2.2)	36 (1.4)		NA	NA	
Highest educational degree earned						
Bachelor’s	297 (22.0)	630 (25.4)	<.001	21 (29.6)	68 (34.2)	.77
Graduate or higher	553 (41.0)	827 (33.3)		47 (66.2)	123 (61.8)	
Other	499 (37.0)	1026 (41.3)		3 (4.2)	8 (4.0)	
Professional history^b^						
Business	649 (26.4)	1312 (36.5)	<.001	32 (22.5)	121 (30.4)	.26
Law	196 (8.0)	331 (9.2)		20 (14.1)	66 (16.6)	
Health care	158 (6.4)	139 (3.9)		9 (6.3)	17 (4.3)	
Public service or politics	919 (37.4)	1095 (30.5)		57 (40.1)	138 (34.7)	
Other	536 (21.8)	715 (19.9)		24 (16.9)	56 (14.1)	

Abbreviation: NA, not applicable.

^a^ Other was reported in the KnowWho dataset for race without additional context.

^b^ Professional history was based on up to 2 most recent professions listed in the KnowWho dataset for each legislator. Because both professions were included, categories were not mutually exclusive and counts and percentages may exceed the number of legislators and sum to more than 100%.

## Discussion

In this national study, there was low representation of female legislators on state and federal health care committees, with some committees having no female members. At the state level, female legislators had, on average, higher educational attainment than men serving on those committees, which may reflect different appointment processes not captured in this analysis.

This study is limited by use of a third-party database and by the absence of information on the mechanisms of committee assignment, which may include seniority systems and self-selection. These findings provide a benchmark for future research on the association of committee composition with health policy outcomes.

## Notes

Supplement.
Data Sharing Statement
