# Supplementary material for: Composition by Sex of State and Federal Legislative Health Committees
Source: JAMA Netw Open. 2026 May 20;9(5):e2613624. doi: 10.1001/jamanetworkopen.2026.13624 (PMC13191375; doi:10.1001/jamanetworkopen.2026.13624)
Supplement: Supplement. — Data Sharing Statement [file jamanetwopen-e2613624-s001.pdf]

Data Sharing Statement

Sung. Composition by Sex of State and Federal Legislative Health Committees. *JAMA Netw Open*. Published May 20, 2026. doi:10.1001/jamanetworkopen.2026.13624

Data
Data available: No
